# The Role of the LY294002 - A Non-Selective Inhibitor of Phosphatidylinositol 3-Kinase (PI3K) Pathway- in Cell Survival and Proliferation in Cell Line SCC-25

**DOI:** 10.31557/APJCP.2019.20.11.3377

**Published:** 2019

**Authors:** Andressa Duarte, Giórgia Gobbi Giórgia Gobbi, Danilo Figueiredo Soave, João Paulo Oliveira Costa, Alfredo Ribeiro Silva

**Affiliations:** 1 *Department of Pathology and Forensic Medicine, Ribeirão Preto Medical School, University of São Paulo,*; 3 *Department of Stomatology (Oral Pathology), Dental School Federal University of Goiás Goiânia, Brazil, *; 2 *Massachusetts General Hospital, Harvard Medical School, Boston, USA. *

**Keywords:** PI3K, AKT, oral squamous cell carcinoma, COX, GLUT1, Bcl-2

## Abstract

The activation of PI3K further activates subsequent regulatory pathways, which are activated via AKT phosphorylation. AKT is closely related to the Bcl-2 family, a protein known to be involved in cell survival. AKT also has a relationship with inflammatory and glycolytic mediators. The present work aimed to evaluate the relationship between the PI3K/AKT pathway, cell survival/proliferation, inflammatory mediators and the glycolytic pathway in oral squamous cell carcinoma. All experiments were performed in the SCC25 oral squamous cell carcinoma cell line. In the presence or absence of PI3K pathway inhibitors, we analyzed the protein expression of pAKT and AKT; X-linked inhibitor of apoptosis protein; Bcl-2-associated death promoter; Bcl-2-like protein two inhibitor; cyclooxygenase 1; cyclooxygenase-2; and glycoprotein-associated glucose transporter 1. For the functional characterization of treated or untreated cells, we also performed matrix invasion assays, cell migration assays, and cell proliferation assays. Our results demonstrated that activation of the PI3K/AKT pathway is directly related to members of the Bcl-2 family and GLUT1, but not the inflammatory mediators COX1 and COX2. Our data suggest that the PI3K/AKT pathway is related to cell survival and proliferation in oral squamous cell carcinoma through its interaction with Bcl-2 family members.

## Introduction

Cancer is a public health problem, with 21.4 million new cases and 13.2 million deaths estimated to occur in 2030 (Ferlay et al., 2015). Cancer of the oral cavity is the 11th most common malignancy in the world, with approximately 90% of oral tumors subclassified as oral squamous cell carcinoma (SCC) (Ghantous et al., 2015). In most countries, the proportion of oral cancer is higher in men than in women(Ghantous et al., 2015).

The molecular biology of head and neck carcinoma is very complex and develops from the dysfunction of several interrelated pathways. There is no mechanism or molecule alone that is responsible for the carcinogenesis (Glazer et al., 2009). In recent years, the PI3K (phosphatidylinositide 3-kinase)/AKT (protein kinase B) signaling pathway has been shown to be involved in many types of cancer in humans, with a role not only in the development of tumors but also in the potential response of the tumor to the treatment. Thus, a better understanding of the PI3K/AKT signaling pathway may improve prognostic capacity and accuracy in treatment.

The enzyme PI3K and the downstream AKT protein were initially identified by their activity associated with various oncoproteins, growth factor receptors, and events associated with the cell cycle (Wymann and Pirola, 1998; Utermark et al., 2012). The first protein described as the target of AKT protein kinase was glycogen synthase kinase 3 (GSK-3), an important enzyme involved in glycogen metabolism (Cross et al., 1995). Currently, several other proteins have been described as targets for phosphorylation and consequent activation or inhibition by AKT, including anti-apoptotic and pro-apoptotic proteins such as the X-linked apoptosis inhibitor (XIAP) (Dan et al., 2004) and BCL-2 associated death promoter (BAD) (Harada et al., 2001). AKT can also interact with glucose transporter 1 (GLUT1) such that AKT activity induces GLUT1 expression on the cellular plasma membrane, where it exerts its glucose transport function (Phadngam et al., 2016b). With regards to inflammatory mediators, the PI3K/AKT pathway is directly related to several inflammatory diseases (Chen et al., 2016).

LY294002 is a chemical compound that is a potent inhibitor of numerous proteins and a strong inhibitor of PI3Ks (Semba et al., 2002). Although it is widely used in the PI3K/AKT, LY294002 it is generally considered a non-selective search tool for PI3K (Gharbi et al., 2007).

BAD is a pro-apoptotic protein of the B-cell lymphoma 2 family (BCL-2) that induces apoptosis by inhibition of the anti-apoptotic proteins. BAD is inactivated by the phosphorylation of a serine residue (Ser136) by AKT, inhibiting its pro-apoptotic functions (Harada et al., 2001; Bergmann, 2002; Chen et al., 2016; Phadngam et al., 2016a). Bcl-w (also known as BCL2L2) is also a member of the Bcl-2 family and is part of the anti-apoptotic group of proteins. It has previously been shown that AKT activity can promote apoptosis through the down-regulation of the Bcl-w protein (Garofalo et al., 2008).

XIAP is the most important member of the inhibitor of apoptosis (IAP) family of proteins, being responsible for inhibiting cellular apoptosis through its ability to bind to caspases 3, 7 and 9 (Deveraux et al., 1997). AKT phosphorylates XIAP at a serine residue (Ser86), and this post-translational modification implies inhibition of XIAP degradation, preventing cells from activating pro-apoptotic factors (Dan et al., 2004).

GLUT1 is a facilitative carrier that plays a major role in glucose internalization. GLUT1 resides in vesicles located in the Golgi complex and is translocated to the plasma membrane after activation of the PI3K/AKT pathway. In the proliferation of cancer cells, which requires high amounts of glucose for their metabolism, GLUT1 is permanently expressed on the plasma membrane. This phenomenon is associated with abnormal activation of the PI3K/AKT pathway (Phadngam et al., 2016b).

Several studies have established PI3Ks as a new therapeutic target, and they are also supposedly involved in the complex pathophysiology of inflammatory diseases and several other diseases. PI3Ks are recognized as participating in inflammatory cellular responses, modulating the growth, development, and proliferation of various immune cells; thus, they affect the release of various cytokines and other inflammatory mediators (Vyas and Vohora, 2016). However, there are no reports of the involvement of PI3Ks with cyclooxygenases (COX).

COX enzymes comprise two isoforms, COX1 and COX2, and play a central role in the biosynthesis of lipid biological mediators, called prostanoids. The COX1 enzyme plays a physiological role, while COX2 is an isoform induced by inflammation. COX2 catalyzes the synthesis of prostanoids, including prostaglandin E2 (PGE2), a major mediator of inflammation and angiogenesis. COX2 is highly expressed in cancer cells and it is associated with the progressive growth of tumors, as well as the resistance of cancer cells to conventional chemotherapy and radiotherapy (Pang et al., 2016). However, there are no studies in the literature showing a direct relationship between the activation of the PI3K/AKT pathway and the expression of COX1 and COX2.

Since the PI3K/ATK pathway is directly related to cell survival as well as the inhibition of apoptotic proteins, inflammatory mediators and glucose transport, this pathway is commonly overactivated in tumors. Currently, several PI3K/AKT pathway component inhibitors have been the subject of research and testing for treatment in different types of cancer (Hennessy et al., 2005). The activation of PI3K occurs in breast, ovary, pancreas, esophagus, and thyroid tumors, among others (Arcaro and Guerreiro, 2007). Although the role of PI3K in tumor proliferation and progression has been characterized, there is little evidence of its role in SCC. The identification of cell characteristics in SCC that express PI3K as well as the relationship of this pathway with the inhibition of pro-apoptotic molecules, inflammatory mediators and glucose transporters may provide us with evidence for the function and regulation of the PI3K pathway in terms of survival, migration and cell invasion. 

## Materials and Methods

*Cell Culture and Reagents*

Cells of the human oral squamous cell line SCC-25 (Cell Bank of Rio de Janeiro, Brazil) were cultured in DMEM:F-12 medium. The media was supplemented with 10% fetal bovine serum and 1% penicillin-streptomycin (Invitrogen, Carlsbad, CA). The cells were maintained as a monolayer in a tissue culture incubator at 37°C with 5% CO_2_ and subcultured every 3–4 days to maintain exponential growth. LY294002 and AKT IV were purchased from Calbiochem (Darmstadt, Germany). ABT-737 was purchased from Abcam (Cambridge, UK).

*Cell growth assay (MTT)*

SCC-25 (2.104) cells were seeded in 96-well plates and cultured in 10% FBS medium for 48 h, in the presence of different treatments. Cells were harvested, 3-(4,5-dimethylthiazol-2-yl)-2,5-diphenyltetrazolium bromide (MTT, 0.5 mg/ml in normal media) was added, and the cells were incubated for 4 h; then, 50 µl HCl and 0,04 M isopropanol were added, and the optical density of 570λ value was detected. 

*Wound-Healing Assay*

SCC-25 cells (1x105) were grown in 12-well plates and allowed to reach confluence. A uniform scratch was made down the center of the well using a sterile micropipette tip, followed by washing with phosphate-buffered saline (PBS) to remove the non-adherent cells. The following drugs were added: Ly29002, AKT IV, and ABT-737. DMSO was used as the drug vehicle in the controls. Digital images of the wound were obtained every 48 h at 10x magnification (Axiovert 200M; Carl Zeiss, Inc., Oberkochen, Germany). The horizontal distance between both sides of the wound was measured. 

*Invasion Assay*

First, the upper compartment of an 8-µm transwell (6.5 mm diameter; Millipore, Massachusetts, USA) was coated with Matrigel diluted with serum-free DMEM:F-12 medium (1:1 dilution) before starting the assay. SCC-25 cells (4x10^5^ cells) were resuspended in serum-free DMEM: F-12 medium and placed in the upper compartment of the transwell. The lower compartment was filled with DMEM: F-12 medium supplemented with 10% FBS. After 48 h, the filter was washed with PBS and fixed with paraformaldehyde. The migrated cells on the filter membrane were stained using DAPI (4’,6-diamidino-2-phenylindole). Each assay was conducted at least three times, and three random fields from a 20X magnification were analyzed for each filter membrane.

*Immunoblotting*

The protein samples were heated to 95°C for 5 min and subjected to SDS–PAGE on 10%-12% acrylamide gels, followed by transfer to nitrocellulose membranes with a Bio-Rad transfer unit (Bio-Rad). The membranes were then blocked for 1 h in Tris-buffered saline with 0.01% Tween 20 (TBS-T) with 5% nonfat dry milk, after which they were incubated overnight with primary antibody in TBS-T with 2% bovine serum albumin (BSA), followed by 1 h incubation with horseradish peroxidase-conjugated anti-mouse or rabbit antibody. The blots were developed with an enhanced chemiluminescence kit (GE Healthcare; Little Chalfont, United Kingdom), Protein levels were quantified by density analysis using Quantity One software (Bio-Rad, Hercules, CA). GAPDH antibody (Sigma–Aldrich) was used as a protein loading control. 

*Statistics*

The results represent the means ± SEM of at least three separate experiments. Differences between the means were determined using a one-way analysis of variance (ANOVA), which was followed by Dunnett’s test for multiple comparisons. The differences were considered significant at P < 0.05. GraphPad Prism 6 software (GraphPad Software) was used for the statistical analysis.

## Results

*The SCC-25 cell line has active PI3K and AKT, which influences the proliferation of these cells*

**Figure 1:**
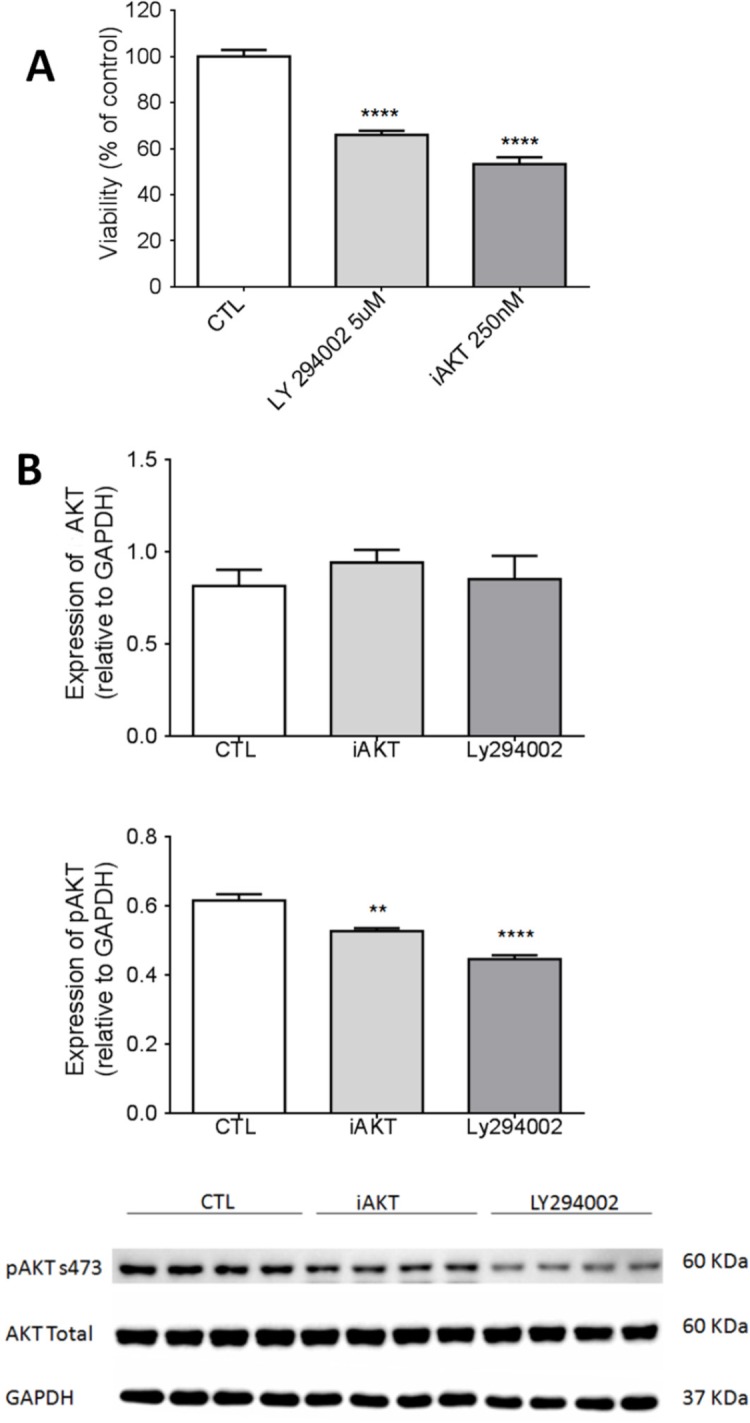
The PI3K/AKT Pathway is Directly Related to SCC-25 Viability. A, Cell line of oral squamous cell carcinoma, SCC-25, treated with Ctl (DMSO), Ly 294002 (5 μM), or iAKT (25 μM). Cell proliferation was assessed in culture after 48 hours of treatment by MTT assay. The results were analyzed by ANOVA followed by Dunnett’s test and are expressed as the mean ± SEM (**** P <0.0001; n = 5); B, SCC25 cells treated for 48 hours with Ctl (DMSO), LY204002 (5 μM), or iAKT (25 μM) and evaluated by western blot for pAKT and total AKT levels. The results were analyzed by ANOVA followed by Dunnett’s test (** p <0.1, *** p <0.001; n = 4)

We performed the MTT assay at concentrations of 250 nM iAKT (AKT inhibitor) and 5 μM LY294002 for 48 h. A decrease in SCC-25 cell viability was observed after the addition of iAKT or LY294002 (Figure 1A). Since we observed the inhibition of SCC-25 cell proliferation after the addition of iAKT and LY294002, we confirmed this observation by analysis of protein expression by the western blot technique to see if the same dose/time of treatment would be effective in blocking AKT phosphorylation. We observed that both inhibitors, iAKT and LY 294002, reduced the phosphorylation of AKT without affecting total levels of AKT (Figure 1B). 

*PI3K/AKT inhibition is related to viability and glycolysis, but not inflammatory mediators*

**Figure 2:**
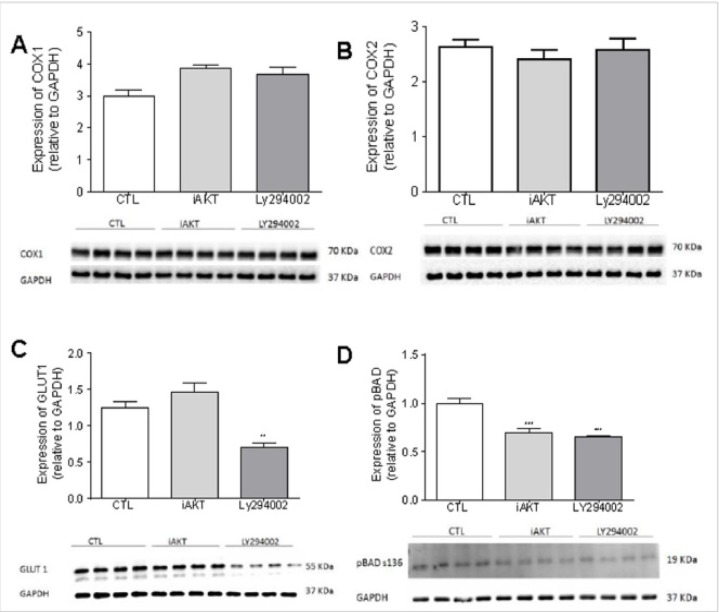
The Effect of PI3K / AKT Blockade on COX, GLUT1 and BAD. A, SCC25 cells treated for 48 hours with Ctl (DMSO), LY204002 (5 μM), or iAKT (25 μM) and evaluated for COX1 levels by western blot. The results were analyzed by ANOVA followed by Dunnett’s test. B, SCC25 cells treated for 48 hours with Ctl (DMSO), LY204002 (5 μM), or iAKT (25 μM) and evaluated for COX2 levels by western blot. The results were analyzed by ANOVA followed by Dunnett’s test. C, SCC25 cells treated for 48 hours with Ctl (DMSO), LY204002 (5 μM), or iAKT (25 μM) and evaluated for GLUT1 levels by western blot. The results were analyzed by ANOVA followed by Dunnett’s test. D, SCC25 cells treated for 48 hours with Ctl (DMSO), LY204002 (5 μM), or iAKT (25 μM) and evaluated for pBAD levels by western blot. All western blot results were analyzed by ANOVA followed by Dunnett’s test (** p <0.1, *** p <0.001; n = 4)

In the present work, we demonstrated that, in SCC-25 oral squamous cell carcinoma cells, the PI3K pathway and the proteins COX1 and COX2 have no direct relation (Figure 2). We showed that both drug treatments reduced BAD phosphorylation (Figure 2D), suggesting that these cells have lost anti-apoptotic protection. 

*Inhibition of Bcl-2 leads to reduced viability of SCC-25 cells*

**Figure 3:**
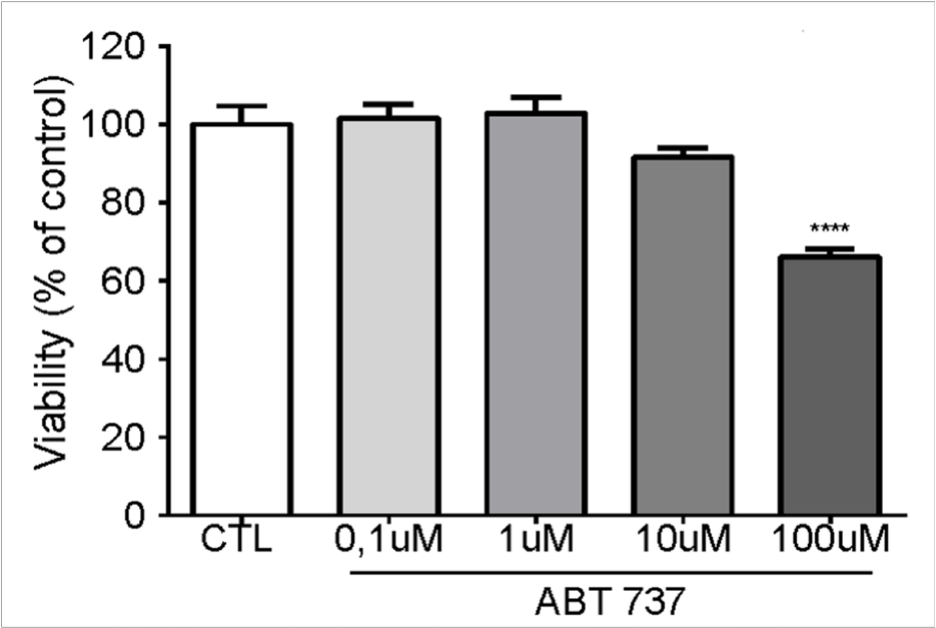
The Inhibition of Bcl-2 Decreases the Cellular Viability of SCC-25 Cells. SCC-25 cells treated with Ctl (DMSO) or ABT 737 (0.1 µM, 1 µM, 10 µM, and 100 µM). After 48 hours of treatment, cell proliferation was assessed by MTT assay. The results were analyzed by ANOVA followed by Dunnett’s test(**** p <0.0001; n = 5)

The dose-response curve was performed in which the cells were treated and incubated for 48 hours with doses of 0.1 μM, 1 μM, 10 μM, and 100 μM ABT-737. We observed a significant reduction in viability when the cells were treated with 100 μM of the inhibitor, and for that reason that dose was used in the following experiments (Figure 3). 

*Inhibition of PI3K decreases the migration, while the inhibition of Bcl-2 influences the migration and invasion of SCC-25 cells*

**Figure 4:**
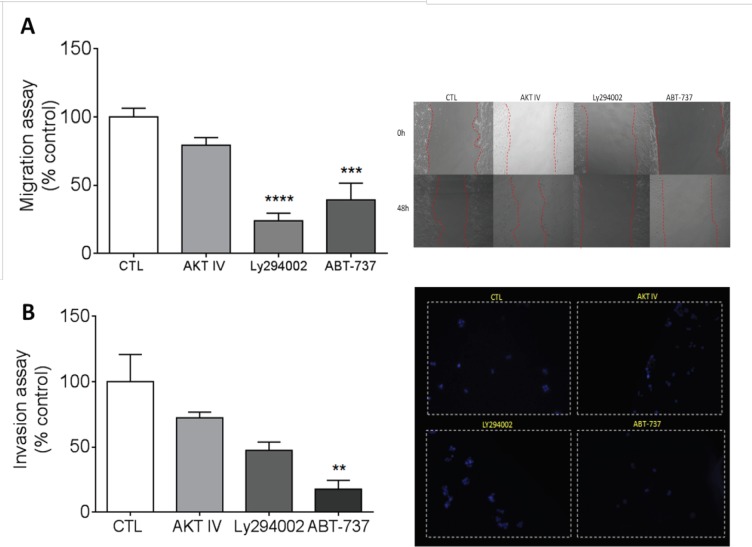
The Inhibition of PI3K, AKT or Bcl-2 in Cell Migration and Invasion. A, To calculate migration/invasion in the presence of inhibitors, the transwell was collected, and the nμMber of invading cells was analyzed. This quantification was performed by counting the cell nuclei stained by DAPI. The results were analyzed by ANOVA followed by Dunnett’s test and are expressed as the mean ± SEM (** p <0.1; n = 4). B, After the cells reached confluence, a lesion was made through the cell monolayer with a 200 μl pipette. The specific fields were imaged at the time of wounding and 48 hours later. The results were analyzed by ANOVA followed by Dunnett’s test and are expressed as the mean ± SEM (** p <0.1; ***p <0.001; **** p <0.0001; n = 4).

The migratory behavior of the cells was analyzed via the migration assay, which was performed in a 48-well plate, and the plate was photographed shortly after the time of the lesion (0 hours) and 48 hours after the time of the lesion. The analysis was carried out in the presence or absence of inhibitors. Moreover, it was observed that the inhibition of PI3K (LY294002) and Bcl-w (ABT-737) significantly inhibited cell migration (Figure 4A). 

## Discussion

Evidence suggests that one of the major functions of the PI3K/AKT pathway is to promote cell growth and survival as well as to block apoptosis (Wymann and Pirola, 1998; Song et al., 2005; Utermark et al., 2012). We first assessed whether inhibition of the PI3K/AKT pathway would inhibit the proliferation of the SCC cell line SCC25. The evaluation of cell viability was performed using MTT methodology (Sigma Aldrich) (Denizot and Lang, 1986). The assay was performed in a 96-well plate containing 2.104 cells per well initially. We first tested the time and concentration of the PI3K inhibitor LY294002 (data not shown). 

In addition to its role in cell survival, the PI3K/AKT pathway is implicated in inflammation and hypoxia (Inukai et al., 2015; Nandipati et al., 2017). We next evaluated the inflammatory axis by analyzing the expression of COX1 (cyclooxygenase-1) and COX2 (cyclooxygenase-2), enzymes responsible for the formation of prostanoids at the hypoxic/glycolytic surface, by analyzing the expression of Glut1 (glucose transporter 1), a membrane-specific glucose transporter. We observed that the PI3K/AKT pathway does not interact with COX1 and COX2 (Figure 2A and 2B), whereas Glut1 expression is decreased after treatment with LY294002 (Figure 2C). 

COX2, an enzyme associated with inflammation, plays a key role in tumor initiation in tissues subject to chronic inflammation (Williams et al., 1999; Subbaramaiah and Dannenberg, 2003). While overexpression of COX2 is both a signature and a mediator of tumor progression and metastasis in a variety of epithelial cancers (Harris et al., 2006), there are no data in the literature that demonstrate a direct effect of the PI3K/AKT pathway on the COX protein family.

GLUT1 is frequently overexpressed in cancer (Szablewski, 2013), which can be attributed to the limited uptake of glucose, even when insufficient glucose is available due to the continuous growth of the tumor (Barron et al., 2016). Studies have shown that the activation of AKT promotes the expression of GLUT1 at the plasma membrane (Morani et al., 2014). In our study, we don’t observe decrease in GLUT1 expression following AKT blockade; however, treatment with the PI3K inhibitor (LY294002) resulted in a significant decrease in GLUT1. Other proteins have been described as targets of AKT, such as BCL-2-associated death promoter (BAD), a pro-apoptotic protein that is inactivated once it is phosphorylated at the Ser 136 by AKT, a process that results in inhibition of apoptosis (Harada et al., 2001; Bergmann, 2002). Our data shows for the first time in head and neck tumors that, in the absence of PI3K/AKT, BAD is not phosphorylated, suggesting that BAD is phosphorylated by PI3K/AKT also in these tumors, a finding in accordance with the current literature (Datta et al., 1997).

ABT-737 is a small molecule that acts as a selective inhibitor of Bcl-2. Bcl-2 proteins play an important role in cell survival and have been shown to be overexpressed in many tumors . ABT-737 also inhibits Bcl-w and Bcl-x1. Since BAD is a member of the Bcl-2 family, that we showed to be affected by the treatment with PI3K and AKT inhibitors, we next sought to analyze ABT-737 in an isolated manner. While it has been shown that the use of ABT-737 together with another drug or combined with chemotherapy decreases viability in head and neck cancer cells (Li et al., 2009; Kim et al., 2017), we observed that ABT-737 alone can inhibit the proliferation of SCC-25 cells. 

PI3K is responsible for coordinating a diverse range of cellular functions, including proliferation, cell survival and cell migration (Carracedo and Pandolfi, 2008). In our study, LY294002 inhibited the migration activity of SCC-25 cells. Koehler et al. observed no change in the migratory capacity of colorectal cancer cells after treatment with ABT-737 (Koehler et al., 2014), suggesting that the tumor site may interfere with the choice of target treatment.

Invasion is an intrinsic characteristic of cancer cells and preventing it would be a crucial step in restraining tumor progression. The assay was carried out with the aim of analyzing the ability of SCC-25 cells to invade the tissue and to evaluate the ability of the drug studied to contain the invasion. We observed that only the Bcl-2 inhibitor (ABT-737) was able to significantly inhibit cell invasion (Figure 4B), demonstrating the importance of Bcl-2 in the process of tumor cell invasion and indicating that ABT-737 may be a good strategy to restrain invasion. Zhang et al. demonstrated that PI3K/AKT is indirectly involved in invasion through an oncogene that acts directly on PI3K/AKT (Zhang et al., 2018). Our findings showed that the direct inhibition of PI3K or AKT did not influence the migratory process of SCC-25 cells. Future studies should investigate whether targeting the inhibition of PI3K and Bcl-2 may be more effective in treating patients with oral squamous cell carcinoma, which would improve the outcome of these highly resistant tumors.

In conclusion, our study verified that, even though (use of LY294002) PI3K/AKT blockade does not have an impact on COX1 and COX2, its inhibition reduces the expression of GLUT1 and affects the phosphorylation of BAD, a member of the Bcl-2 family. We were also able to show that blocking of Bcl-2 by ABT-727 causes a decrease in the invasion capacity of the tumor cells studied. These findings may have clinical importance, allowing for the identification of new pharmacological targets and resulting in alternative therapeutic regimens based on the inhibition of PI3K or Bcl-2. Despite the relevance of our findings, animal testing and human tumor biopsy analysis are still necessary so that we can suggest a target treatment or even a compatible biomarker.
